# Kidney Disease after Heart and Lung Transplantation

**DOI:** 10.14797/mdcvj.1122

**Published:** 2022-09-06

**Authors:** Carlos M. Zapata, Hassan N. Ibrahim

**Affiliations:** 1 Division of Kidney Diseases, Hypertension and Transplantation, Houston Methodist Hospital, Houston, Texas, US; 2 Division of Kidney Diseases, Hypertension & Transplantation, McGovern Medical School, University of Texas Health Science Center at Houston, Houston, Texas, US

**Keywords:** kidney disease, CKD, heart transplantation, lung transplant, AKI, solid organ transplant, simultaneous transplant, post-transplant CKD

## Abstract

Chronic kidney disease (CKD) is not only common after lung and heart transplantation but also is associated with increased morbidity and mortality due to multiple pre-, peri- and post-transplant factors. While the exact incidence of CKD in this population is not well-defined, it seems to have gradually increased over the years as older recipients are more frequently considered. The increasing success of the procedure and expanding transplant candidate pool has allowed many with comorbid conditions to receive a transplant, which was considered prohibitive in the past. This review presents risk factors that have been linked to CKD as well as interventions that may help alleviate this serious problem. The impact of pretransplant renal function and the overexaggerated role of chronic nephrotoxicity of calcineurin inhibitors is discussed in detail. Until the exact pathophysiology of kidney disease is better understood, there is a dire need to expand the research agenda beyond observational studies.

## Introduction

Chronic kidney disease (CKD) is defined as an estimated glomerular filtration rate (eGFR) < 60 mL/min/1.73m² lasting ≥ 3 months. It affects 15.8% of lung transplant recipients, 10.9% of heart transplant recipients, and 6.9% of combined heart-lung recipients in the first 5 years after transplantation.^1^ In the most detailed study of the evolution of kidney function in lung and heart-lung transplant recipients, Canales et al. demonstrated these important findings after 9 to 222 months of follow-up:^2^

1. Pretransplant GFR was 96.3 ± 34.5 mL/min/1.73m^2^.
2. Most of GFR loss occurred in the first year.
3. 55% of recipients had doubling of serum creatinine, ie, lost half of their baseline GFR.
4. 7.3% developed kidney failure and required dialysis or kidney transplantation.
5. Older age, lower GFR at 1 month, and cyclosporine use (rather than tacrolimus) were associated with a higher likelihood of developing kidney failure.
6. 65% died during the follow-up but the incidence of kidney failure was accounted for in a competing risk analysis.

## Pretransplant renal function

Multiple autopsy studies of lung and heart transplant candidates dying on the waiting list show extensive glomerulosclerosis. In those awaiting heart transplantation, significant arteriolar hyalinosis is almost universally present.^3^ A reliable measure of true kidney function should be obtained in all transplant candidates. Most transplant candidates are chronically ill and may have a poor nutritional status, significant weight loss, edema, and decreased muscle mass.^4^ In addition, many afflicted with chronic lung disease have received corticosteroids, chronically leading to loss of muscle mass that results in a lower serum creatinine and therefore an under appreciation of true kidney function. Utilization of 24-hour creatinine clearance to measure kidney function is commonly practiced by many transplant centers. This method, however, overestimates true GFR and is quite cumbersome.^5^ We believe that all candidates should undergo GFR measurement using iohexol, iothalamate, or radioisotope-based methods.

Certain cardiac and pulmonary diseases are associated with CKD and should be taken into consideration. Those with bronchiectasis, pulmonary fibrosis and hypertension, cystic fibrosis (CF), or α-1 antitrypsin deficiency have a higher incidence of CKD. For example, those with cystic fibrosis are prone to nephrocalcinosis from altered intestinal oxalate handling. Moreover, the majority of patients with cystic fibrosis have diabetes and require frequent antibiotics against Gram-negative organisms, many of which can be nephrotoxic. Patients with α -1 antitrypsin deficiency may have antineutrophil cytoplasmic antibody-related kidney disease.^6^ Effective circulating volume in the setting of heart failure is reduced and can result in reduced renal blood flow and GFR, which also should be measured directly in this population. Cystatin C is a freely filtered glycoprotein that can be used to estimate GFR. It is less affected by muscle mass but is prone to be altered by inflammation and use of statins. Despite these limitations, it is superior to using creatinine-based methods of estimating GFR.^4^ Lastly, a kidney biopsy should be done on any candidate with proteinuria to determine etiology and severity of disease in order to determine candidacy for receiving a kidney transplant.

## Recipient demographics and comorbidities associated with CKD

Comorbidity burden, African American ethnicity, and older age at transplant are associated with increased risk of CKD after lung and heart transplantation. Hypertension is present in as many as 90% of heart transplant and 60% to 70% of lung transplant recipients. Level of blood pressure at 1 year also has been identified as an independent risk factor for faster GFR decline 1 to 5 years after lung and heart transplantation.^7^ In addition, type 2 diabetes is present in many of these recipients, particularly those with CF, which undoubtedly is associated with structural renal disease.

### Acute Kidney Injury

**Figure 1:**
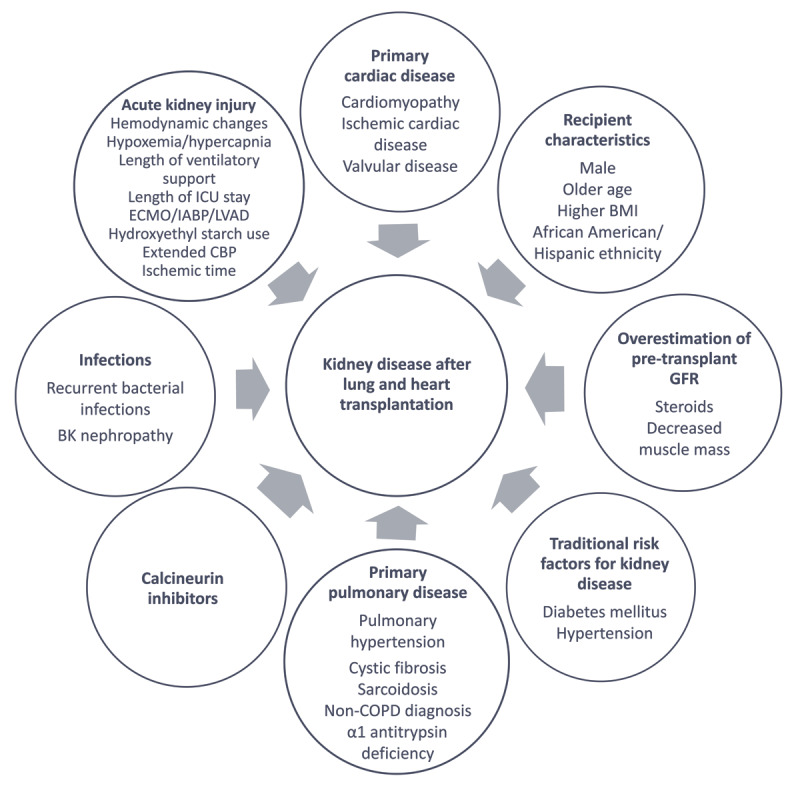
Contributors to kidney disease after heart and lung transplantation. ICU: intensive care unit; ECMO: extracorporeal membrane oxygenation; IABP: intra-aortic balloon pump; LVAD: left ventricular assist device AKI: acute kidney injury; CBP: cardiopulmonary bypass; BMI: body mass index; GFR: glomerular filtration rate; COPD: chronic obstructive pulmonary disease; HTN: hypertension

Acute kidney injury (AKI) in the immediate postoperative period is a powerful predictor of post-transplant CKD and death.^2,8^ In fact, lung transplant recipients who develop AKI and require dialysis early after transplant have an almost 100% mortality at 2 years.^8^ Major alteration in hemodynamics, duration of mechanical ventilation, need for vasopressor support, use of cardiopulmonary bypass, extracorporeal membrane oxygenation, requirement for intra-aortic balloon pump, and left ventricular assisted device have been identified as possible risk factors for AKI development (Figure 1).

In a large meta-analysis by Ploypin et al. of 26 cohort studies comprising 40,592 recipients, 52.5% developed AKI and 9.3% had severe AKI requiring renal replacement therapy.^6^ Frequency of AKI after heart transplant recipients is roughly similar. In a retrospective cohort study of 597 patients by Git et al., 76% of recipients developed AKI and 5% developed severe AKI requiring renal replacement therapy.^9^ Also, multiple case reports of AKI following non-renal transplantation point to acute oxalate nephropathy.^10^ It is presumed that prolonged exposure to antibiotics interferes with colonic flora and therefore leads to enteric hyperoxaluria.

### Role of Calcineurin Inhibitors in CKD after Heart and Lung Transplant

There are substantial differences between renal transplants and non-renal solid organ transplants: mainly the alloimmune response to the “non-self” renal allograft. Therefore, in non-renal solid organ transplantation, acute rejection phenomena that are key determinants of renal function are absent. Moreover, it has been suggested that cyclosporine (CsA) stimulates sympathetic nerve activity in native kidneys, playing a role in the acute nephrotoxic effects of CsA by increasing renal vascular resistance and thereby causing an acute decline of the GFR.^11^ Transplanted kidneys lack sympathetic innervation, so the potentiation of those nephrotoxic effects by calcineurin inhibitors (CNI) is not observed through sympathetic upregulation in renal allografts.^12^ No surveillance kidney biopsies are performed in non-renal transplant recipients and therefore no data is available on the evolution of CNI-related histological changes. The initial report linking CNI to renal damage in non-renal transplants was by Myers et al., who pointed out that long-term CsA use in heart transplant recipients may lead to irreversible renal dysfunction.^1^ Of note, the targeted trough CsA level in that study was very high (300-350 ng/mL). Ojo et al. studied the cumulative incidence of CKD in a national cohort of solid non-renal transplant recipients and found that the use of CNI was associated with increased relative risk for kidney dysfunction but was not a risk factor for CKD in the multivariate analysis.^1^

To understand the “true” contribution of CNI to renal dysfunction, we appraise the evidence from randomized trials that avoided CNI use completely, compared low versus high CNI levels, and lastly converted recipients from CNI to alternative agents:

#### 1. CNI-Sparing Protocols

The earlier reports of cyclosporine or tacrolimus (TAC) use along with sirolimus (SRL) in order to minimize CNI showed a decrease in acute rejection incidence compared to a regimen of CsA, azathioprine, and glucocorticoids.^13^ Conversely, later reports showed that the use of SRL in combination with CNI is associated with inferior renal graft survival and renal dysfunction compared with CsA or TAC with mycophenolate mofetil (MMF) and corticosteroids.^14^ For that reason, the use of CNI combined with SRL is generally considered in patients on an individualized basis due to the aforementioned overall inferior graft outcomes.

#### 2. CNI Avoidance and Minimization Protocols

CNI-free protocols do not appear to improve long-term kidney transplant outcomes. The ELITE-Symphony study was a randomized prospective trial that compared CNI avoidance and minimization strategies by randomizing kidney transplant recipients to low-dose SRL, low-dose TAC, low-dose CsA, or standard-dose CsA. Renal allograft function was better and biopsy-proven AR rates were significantly lower in the low-dose TAC group compared to all other treatment groups.^15^ Furthermore, allograft survival was better with low-dose TAC compared to standard-dose CsA and low-dose SRL. CNI avoidance with low-dose SRL failed to show improvement in renal function, and biopsy-proven AR rate and graft survival were significantly worse than low-dose TAC.

Many other trials also have shown that CNI avoidance protocols did not confer a benefit on GFR or allograft histology in kidney transplant recipients treated with SRL.^16,17^ In a randomized trial of complete avoidance of CNI, Larson et al. compared 81 kidney transplant recipients who received SRL-MMF-prednisone to 83 who received TAC-MMF-prednisone as maintenance immunosuppression with a mean follow-up of 33 months.^16^ The 1-year patient and graft survival were similar in both groups, and there was no difference in iothalamate GFR between the TAC and SRL groups at 1 or 2 years. Interestingly, there was no difference in interstitial, tubular, or glomerular changes at 1 year by the Banff chronicity criteria scoring. In the Orion Study, Flechner et al. randomized kidney transplant recipients to three groups: SRL-TAC followed by TAC elimination at 13 weeks, SRL-MMF, and TAC-MMF. The SRL-MMF group had high biopsy-proven AR and was terminated early by the sponsor. The SRL-based regimens were associated with poorer outcomes in kidney transplant recipients.^18^

Another randomized trial showed that conversion from TAC to SRL at 1-month post-transplant in kidney transplant recipients does not halt or improve the progression of chronic changes on protocol biopsies during the first 2 years, even in patients without previous rejection.^19^ More recently, belatacept (a T-cell costimulation blocker) was studied as an alternative to CNI. Two trials compared belatacept with CsA: the BENEFIT trial in recipients of standard-criteria living kidney donors, and the BENEFIT-EXT in recipients of extended-criteria deceased kidney donors.^20^ In the BENEFIT trial, belatacept had similar patient and graft survival rates compared with CsA at 1-year post-transplant. Belatacept-treated patients had higher measured GFR but had a higher incidence of early acute rejection. Importantly, chronic allograft changes were minimally reduced in the belatacept groups and at 5 years showed no difference in chronic allograft nephropathy on biopsy.^20,21^

#### 3. CNI Elimination Protocols

CNI elimination involves the complete withdrawal of CNI from transplant recipients who had been initially placed on CNI. The main trials were the CONCEPT study, Spare-the-Nephron, and CONVERT trial.^22,23,24^ The CNI withdrawal group in the CONCEPT study showed a better renal allograft function but not the Spare-the-Nephron or CONVERT trials.^24^ Late CNI withdrawal in the CONCEPT trial was actually harmful to recipients with proteinuria. The CAESAR trial evaluated CNI minimization and withdrawal strategies by randomizing kidney transplant recipients to low-dose CsA, low-dose CsA with early withdrawal, and standard-dose CsA.^25^ Renal allograft function was similar in all three groups. However, the biopsy-proven rejection rate was higher in the CsA withdrawal group. Taken together, avoiding or minimizing CNI in renal transplant recipients generally results in higher acute rejection rates and no appreciable improvement of GFR or renal histological changes. It also has become clear that CNI minimization or avoidance may trigger humoral immunity. The applicability of these data to lung and heart transplant recipients has not been established.

## Post-transplant factors

Over half of lung and heart transplant recipients reach stage 3 CKD by 1 year and those who survive the first year maintain this degree of GFR depression at 7 years, emphasizing that most renal function loss happens in the first year after transplantation.^2^ Beyond the first year, more traditional risk factors for CKD development emerge. Hypertension is commonly observed post transplantation and CNI are important contributors to post transplantation hypertension, which is often characterized by a low renin and aldosterone state.^2^ Ishani et al. demonstrated that diastolic blood pressure elevation is an independent predictor of progressive kidney disease after lung and heart-lung transplantation.^26^ This observation is consistent with those in nontransplant patients. Blood pressure goals should be ≤ 130/80 mm Hg in general and < 120/80 mm Hg if there is CKD or proteinuria. Angiotensin-converting-enzyme (ACE) inhibitors and angiotensin II blockers are considered first-line therapy in hypertension in the setting of native CKD and also in kidney transplant recipients. The goal of blood pressure target, and which agents are preferred, in heart and lung transplant recipients have not been studied.^27^

BK nephropathy, while rare, has been described in lung and heart transplant recipients and should be considered in the workup of renal dysfunction after lung and heart transplantation.^28^ Moreover, treatment of CMV disease with antivirals, particularly Foscarnet, can lead to significant decline in kidney function.^29^ Lastly, bacterial infections are quite common in these recipients, which may lead to acute tubular necrosis in the setting of severe sepsis and occasionally AKI related to antimicrobial nephrotoxicity.

### Nephroprotective Strategies

There is no agreed-upon guidance for how to best address renal dysfunction in lung and heart transplant recipients. Clinicians are guided by the same principles used by the general CKD population and kidney transplant recipients as outlined in the Kidney Disease Outcomes Quality Initiative. Recommendations include strict blood pressure control (as described) and initiation of ACE-inhibitors or angiotensin II blockers as soon as there is evidence of proteinuria and/or a diagnosis of diabetes mellitus. Blood pressure goal target < 120/80 mm Hg for those with eGFR < 60 mL/min, dietary salt restriction to < 2g per day, and bicarbonate supplementation in patients with bicarbonate < 22 mmol/L should be considered.^30,31^ Multidisciplinary discussions frequently consider lowering CNI exposure to the minimum level for effective immunosuppression, but the implication for acute rejection is substantial.^32^ In those with advanced CKD, prompt placement on the kidney transplant waiting list and consideration for live donor transplant is critical because the mortality of these recipients on dialysis is quite high.

## Conclusion

Kidney disease after lung and heart transplantation is common and is associated with morbidity and mortality. AKI shortly after transplantation, especially if it requires dialysis, is perhaps the most powerful predictor of future CKD and kidney failure. Most renal function loss occurs in the first-year post transplantation and is more common in those with nonchronic obstructive pulmonary disease diagnosis. Strict blood pressure control (< 130/80 mm Hg), tight glycemic control, and avoidance of nephrotoxic medications are the general guiding principles in these recipients. Avoidance and minimization of calcineurin inhibitors is not recommended and may increase risk of rejection.

## Key points

- As more older patients are considered for lung and heart transplantation and experience continued improvement in long-term outcomes, we are likely to see more kidney disease in this population.
- A notable number of recipients have significant renal impairment prior to transplantation, which is missed if glomerular filtration rate (GFR) is estimated or assessed by 24-hour creatinine clearance; therefore, measured GFR should be done in all candidates.
- Almost one out of five lung and heart transplant recipients develops kidney failure in the first 10 to 15 years after transplantation.
- Older age, acute kidney injury shortly after transplantation, and nonchronic obstructive pulmonary disease diagnosis are prominent risk factors for kidney disease development.
- It is doubtful that use of calcineurin inhibitors is a major contributor to chronic kidney disease after transplantation.
- Lung and heart transplant recipients with progressive kidney disease should be considered for preemptive kidney transplantation since their mortality on dialysis is elevated.
